# Seroprevalence of Hepatitis B virus and Hepatitis C virus among blood donors in Nyala, South Dar Fur, Sudan

**DOI:** 10.1186/1743-422X-6-146

**Published:** 2009-09-23

**Authors:** Mustafa Abdalla Ali Abou, Yassir Mohammed Eltahir, Abdelwahid Saeed Ali

**Affiliations:** 1 Department of Laboratories Administration, State Ministry of Health, South Dar Fur State, Nyala, Sudan; 2 UNAMID Level 1 Clinic, Nyala, South Dar, Sudan; 3 Department of Preventive Medicine & Veterinary Public Health, Facualty of Veterinary Science, University of Nyala, Nyala, Sudan; 4 Department of Preventive Medicine, Faculty of Veterinary Medicine, University of Khartoum, Khartoum, Sudan

## Abstract

The objective of this study was to determine the seroprevalence of hepatitis B virus (HBV) and hepatitis C virus (HCV) infections and the possible risk factors among blood donors in Nyala, South Dar Fur State of western Sudan, which has never been studied before. A total of 400 male blood donors were tested for the detection of HBsAg and anti-HCV antibodies, (6.25%) were found reactive for HBsAg and (0.65%) were reactive for anti-HCV antibodies. The highest seroprevalence (30.8%) was found in those between 19-24 and 37-42 years for HBsAg, whereas it was (50%) in those between 31-36 years for anti-HCV antibodies. Unprotected sexual activities (20%) was the most apparent predisposing risk factor for both HBV and HCV seroreactors, followed by razor sharing (13.3%), parenteral drug injections (10%), history of migration to Egypt and alcoholism (6.6%) for each, tattooing and surgical procedures (3.3%) for each and (36.6%) were not aware for their condition. Serum alanine aminotansferase (ALT) was elevated in (30.7%) of HBV seroreactors and in (50%) of HCV seroreactors. Serum albumin was reduced in (23.1%) HBV and in (50%) HCV seroreactors. The study concluded that the seroprevalence of HBV and HCV was in an intermediate and low rates respectively and unprotected sexual activities was the major risk factor for infection in the population studied.

## Introduction

Hepatitis B virus (HBV) and hepatitis C virus (HCV) are the two major causes of chronic liver disease worldwide. Both viruses are hepatotrophic, but not directly cytopathic and elicit progressive liver injuries resulting in the end-stage liver disease unless effectively eradicated [1,2]. In Sudan, the incidence of hepatocellular carcinoma (HCC) is high and increasing, in one study conducted in 1996-1998 among 150 HCC patients, indicated that HBV and HCV are important risk factors of HCC in Sudan [3]. Both human viral infections can be transmitted by various routes, i.e. blood and blood products (e.g. blood transfusion), sexual, oral, vertical and horizontal transmission [4-6]. The well known and routinely used screening serologic techniques Enzyme Linked Immunosorbent Assay (ELISA) and Immunochromatographic test (ICT) were both employed to detect HBsAg and anti-HCV antibodies among male blood donors attending Nyala Teaching Hospital. Demographic studies using interviewing questionnaire were also used to illustrate the possible risk factors for both viral infections. The basic epidemiological data for these viruses might be of great importance to the programme managers and health planers, so as to initiate the screening package in the blood banks. A little proper published data of the epidemiology of viral hepatitis is available in Sudan in general and nothing in Dar Fur region particularly. Thus the current study aimed to determine the seroprevalence and the possible risk factors for HBV and HCV among blood donors in Nyala.

## Materials and methods

This study was conducted during the period from May to July 2007, as a hospital based study in the city of Nyala, South Dar Fur State, Sudan. The reason for selecting this area was that no previous studies were done to investigate the seroprevalence of both human viral infections. The population of Nyala area is approximately 1.500.000. Subjects were apparently healthy male blood donors (n = 400) attending Nyala Teaching Hospital and randomly selected as study subjects and enrolled after interviewing questionnaire include the demographic information (age, sex and residence), history of previous blood transfusion and other possible risk factors (e.g. unprotected sexual activities). Five ml of blood were drawn from each subject; sera were separated, aliquoted, labeled within two hours of collection and stored at -70°C. Serum samples initially tested for HBsAg and anti-HCV antibodies with immunochromatographic test (ICT) from Advanced Quality, then screened with a 3^rd ^generation ELISA, Equi-HBsAg and EIAgen anti-HCV antibodies from Equibar and Adalits respectively.

### Statistical Analysis

Processing and analysis of data were performed by means of the statistical package for social sciences (SPSS-PC version 10.0, computer software). Statistical significance was taken as (P ≤ 0.01) for T-student and Chi-square tests.

## Results

**Figure 1:**
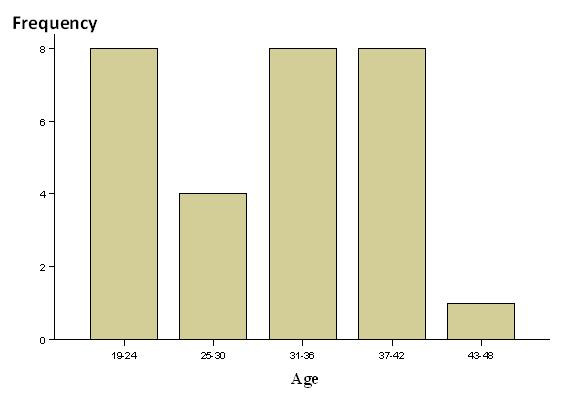
**Age groups of the HBV and HCV seroreactors (vertical axis shows the frequency whereas the horizontal axis shows the age group per year)**.

A total of 400 subjects were included in the study, with a mean age of 33 years and an age range of 18-49 years, all of them were males. The seroprevalence of HBV revealed from ICT results was (6%), whereas it was increased to (6.5%) by ELISA procedure ran on the same serum samples. The mean seroprevalenve of HBV calculated from both tests was (6.25%). On the other hand the seroprevalence of HCV revealed from ICT results was (0.3%), whereas it was increased to (1%) by ELISA procedure ran on the same samples. The mean seroprevalence of HCV calculated from both tests was (0.65%). Some selected liver function tests e.g. serum alanine aminotransferase (ALT) and serum albumin were done to HBV and HCV reacted samples, ALT was elevated in (30.7%) of HBV reacted samples while it was elevated in (50%) of HCV reacted samples. Serum albumin was reduced in (23.1%) of HBV reacted samples while it was also reduced in (50%) of HCV reacted samples. The highest percentage (30.8%) of HBV reacted samples were aged within the age group 19-24 and 37-42 for each. On the other hand the highest percentage (50%) of HCV reacted samples were aged within the age group 31-36. Most (58%) of HBV seroreactors were from Nyala city, while only (15%) were from Kalma camp and (27%) from other South Dar Fur State localities. In contrast (50%) of HCV seroreactors were from Nyala and (25%) from Kalma camp and Beliel locality for each. Unprotected sexual activities (20%) was the apparent predisposing risk factor, followed by razor sharing (13.3%), parenteral drug injections (10%), history of migration to Egypt and alcoholism (6.6%) for each, tattooing and surgical procedures (3.3%) for each and (36.6%) were not aware for their condition (figure 1).

## Discussion

All subjects examined in this study were males, because usually females do not donate blood in Sudan. This is because socially and culturally women are not favored to donate blood either to men or women. Usually men of young or middle age are those who can willingly donate their blood.

Results showed that the seroprevalence of HBV and HCV among blood donors in Nyala was (6.25%) and (0.65%) respectively, this indicates an intermediate HBV seroprevalence [7] and low level of HCV seroprevalence. The study showed that the seroprevalene of HBV is in compliance rate if compared to previous studies conducted in northern and central Sudan (5.1%) and (5.6%) respectively [8,9]. Whereas it was low if compared to southern Sudan (26%)[10]. On the other hand the seroprevalence of HCV among blood donors in Nyala is nearly the same (0.65) as in central Sudan (0.6%)[9], whereas it was a bit lower in comparison to northern Sudan (1.3%), Gezira state (2.2%) and southern Sudan (3%) respectively [8,10,11].

Results showed that the ICT used in this study for HBsAg and anti-HCV antibodies screening was less sensitive when compared with ELISA since additional positive samples were detected by ELISA for both viruses (two for HBV and three for HCV), this could be due to short incubation period of the ICT employed in the study. Characteristically short incubation tests do not detect low affinity or low concentration of antibodies as well the classic type of immunoassays which employ longer incubation times allowing reaction to proceed to completion. It should be noted that the ICT may identify HBsAg and anti-HCV antibodies, negative samples reasonably well, but, because of their short incubation times the assays do not always identify low affinity/low concentration of antibodies. Therefore the potential for false negative results in short incubation test are higher than that in Enzyme Immuno Assay (EIAs) using longer incubation periods and multiple antigens. All over it is clear that antibody to hepatitis B surface antigen (anti HBs) could not be detected at early stage of HBV infection i.e. before three to six weeks post infection and also at late stage three to six months post infection. This could explain why ICT has not detected such samples since it is directed to anti HBs antigen. The same reason could be also attributed to the failure of ICT to detect HCV positive samples. These results obviously showed that the possibility of false negative results could be obtained by ICT which is usually practiced in Nyala Teaching Hospital blood bank as a preliminary screening test and thereby the risk of transfusing infected blood and establishment of new foci of infection exist.

In this study liver damage assessment of the positive samples was performed by the alanine aminotransferase (ALT) and albumin parameters. The ALT is thought to be more specific indicative parameter of hepatocellular damage and its results interpretation reflect the infection status. The ALT is markedly elevated in acute infections, mildly elevated in chronic infections and it is normal in resolved infections [12]. Therefore results showed that eleven of the thirty HBV and HCV positive samples has elevated ALT, seven of them were markedly elevated (ALT ≥ 40 U/I) above the reference value (5-25 U/I) which indicate an acute infection, four were have mild ALT elevation (ALT = 28 to31 U/I) which indicate a chronic infection, whereas the remainder nineteen positive samples with normal ALT level which indicate a resolved infection.

The study showed that serum albumin was reduced below the reference value (3.8-5.1 g/dl) in each of four different HBV and HCV positive samples out of thirty (serum albumin ≤ 3.0 g/dl). In acute liver disease however, there may be little or no reduction in plasma albumin as the biological half-life of albumin is about 20 days and the fractional clearance is therefore low [13]. In contrast in chronic liver disease when large numbers of parenchymal liver cells have been destroyed, synthesis of albumin is impaired. In the absence of other known causes of hypoproteinaemia a plasma albumin concentration below 3.0 g/dl indicates liver damage [14]. More over lower serum albumin and progressive liver disease was also associated with the coexistence of mutant variant of viruses that might have higher pathogenecity and replication ability [15].

In this study the predisposing risk factors of HBV and HCV infections were assessed using the history of the positive blood donors. Unprotected sexual activities was the most apparent predisposing risk factor (20%), followed by razor sharing (13.3%), parenteral drug injections (10%), migration to Egypt and alcoholism (6.6%) for each, tattooing and surgical procedures (3.3%) for each. These results were in accordance with central Sudan (3%) and Gezira state (2.7%) concerning tattooing [9,11] whereas it was much lower than the southern Sudan for HBV (95%) and (43%) for HCV [10]. For parenteral drug injections these results were a bit lower than the Gezira state (18.8%) [11] and much lower for HBV (26.7%) and the same (10.2%) for HCV when compared to Omdurman area [16]. Alcoholism behavior is known to favors viral hepatitis, such behavior reported in this study was in compliance (8%) if compared to North Kordofan state and Gezira state [3]. History of migration to Egypt could be explained as a predisposing risk factor by the fact that, previous studies has shown that the Nile delta of Egypt has one of the highest seroprevalence rates of viral hepatitis infection in the world approaching (20%) [17]. Surgical procedures as a predisposing risk factor in this study was in the same rate when compared to Gezira state (5.1%)[11].

Unprotected sexual activities which was appeared as the most predisposing risk factor (20%) in this study, when compared to previous studies conducted in Sudan, no data was available and this may be due to fact that in Sudan, answering questions relating to sexual activity is confidential or taboo. It should be noted that other unknown or unstudied risk factors like circumcision, public nail clipping and any other cultural behavior that include direct blood contact might contribute the transmission of these viruses.

## Conclusion

The study concluded that the seroprevalence of HBV (6.25%) and HCV (0.65%) was in an intermediate and low rates respectively and unprotected sexual activities (20%) was the major risk factor for infection in the population studied.

## Competing interests

The authors declare that they have no competing interests.

## Authors' contributions

MAAA carried out the entire work of the study. YME supervised the entire work of the study. ASA discussed and approved the study. All authors read and approved the final manuscript.
